# Insights into Chitin-Degradation Potential of *Shewanella khirikhana* JW44 with Emphasis on Characterization and Function of a Chitinase Gene *SkChi65*

**DOI:** 10.3390/microorganisms12040774

**Published:** 2024-04-11

**Authors:** Ling Wang, Ming Xue, Rui Yan, Jiawei Xue, Zhipeng Lu, Chongqing Wen

**Affiliations:** 1 Fisheries College, Guangdong Ocean University, Zhanjiang 524088, China; 2 Guangdong Provincial Key Laboratory of Aquatic Animal Disease Control and Healthy Culture, Zhanjiang 524088, China

**Keywords:** *Shewanella khirikhana*, chitin degradation, chitinase, biochemical characterization

## Abstract

Chitin, a polymer of β-1,4-linked *N*-acetylglucosamine (GlcNAc), can be degraded into valuable oligosaccharides by various chitinases. In this study, the genome of *Shewanella khirikhana* JW44, displaying remarkable chitinolytic activity, was investigated to understand its chitin-degradation potential. A chitinase gene *SkChi65* from this strain was then cloned, expressed, and purified to characterize its enzymatic properties and substrate hydrolysis. Genome analysis showed that, of the 14 genes related to chitin utilization in JW44, six belonged to glycoside hydrolase (GH) families because of their functional domains for chitin binding and catalysis. The recombinant chitinase SkChi65, consisting of 1129 amino acids, was identified as a member of the GH18 family and possessed two chitin-binding domains with a typical motif of [A/N]KWWT[N/S/Q] and one catalytic domain with motifs of DxxDxDxE, SxGG, YxR, and [E/D]xx[V/I]. SkChi65 was heterologously expressed as an active protein of 139.95 kDa best at 37 °C with 1.0 mM isopropyl-β-d-thiogalactopyranoside induction for 6 h. Purified SkChi65 displayed high stability over the ranges of 30–50 °C and pH 5.5–8.0 with optima at 40 °C and pH 7.0. The kinetic parameters *K_m_*, *V_max_*, and *k*_cat_ of SkChi65 towards colloidal chitin were 27.2 μM, 299.2 μMs^−1^, and 10,203 s^−1^, respectively. In addition to colloidal chitin, SkChi65 showed high activity towards glycol chitosan and crystalline chitin. After analysis by thin-layer chromatography, the main products were *N*,*N*’-diacetylchitobiose, and GlcNAc with (GlcNAc)_2–6_ used as substrates. Collectively, SkChi65 could exhibit both exo- and endochitinase activities towards diverse substrates, and strain JW44 has a high potential for industrial application with an excellent capacity for chitin bioconversion.

## 1. Introduction

The polysaccharide chitin, composed of *N*-acetylglucosamine (GlcNAc) residues linked by β-1,4 glycoside bonds, is the second most abundant biomass after cellulose [1]. The annual production of chitin in the aquatic biosphere alone exceeds 10^11^ metric tons; however, this rich resource has not been fully utilized because of its insolubility [2,3]. In nature, chitin is the principal component of the cell walls of fungi, exoskeletons of insects, and shells of crustaceans and certain mollusks [1,4]. Currently, many methods are used to transform chitin into high-value-added derivatives, including chitosan, chitooligosaccharides (COSs), and GlcNAc, which are of considerable interest in medicine, pharmaceutics, agriculture, and biotechnology [5,6].

Relative to chemical procedures for chitin degradation, enzymatic methods are more gradual, with higher efficiencies and fewer environmental hazards; thus, developing excellent chitinolytic enzymes has been the focus of numerous studies [6,7]. Chitinases (EC 3.2.1.14) are synthesized by diverse organisms depending on the physiological function of chitin as a substrate for the producers [8,9]. Microbial chitinases produced by bacteria, archaea, and fungi play crucial roles in chitin degradation and recycling in nature [10,11]. Generally, chitinases are classified as two different types based on their mode of action, i.e., endochitinases, cleaving randomly at internal sites of chitin, generate diverse COSs such as chitotriose and chitotetraose; while exochitinases, including chitobiosidases and β-1,4 *N*-acetylglucosaminidases, catalyze the progressive release of chitobiose from the non-reducing end of chitin and cleave COSs to monomers, respectively [12,13,14].

To date, bacterial chitinases have been mainly assigned to glycoside hydrolase (GH) families 18, 19, and 20 based on amino acid sequence similarity [15]. Members of the GH18 family are separated into three subfamilies, A, B, and C. Chitinases from subfamily A are studied most intensively, with modular structures, including typical domains of (α/β)_8_-TIM barrel, fibronectin type III, and chitin-binding, which play important roles in catalysis, linkage, and binding, respectively [12,16,17]. In addition, a small α + β chitinase insertion domain (CID) presents in the catalytic domain of subfamily A but is absent in subfamily B; thus, the substrate-binding clefts from subfamily A are deeper than those observed in subfamily B [18,19,20].

A variety of chitinases have been characterized in terms of temperature and pH dependency. The pH optima of bacterial chitinases vary over a wide range, such as acidic pH (5.0), neutral pH (7.0–7.5), and basic pH (8.0–9.0) for enzymes from *Paenicibacillus barengoltzii* CAU904, *Bacillus* sp. R2, and *Pseudoalteromonas* sp. DC14, respectively [21,22,23]. Moreover, most reported bacterial chitinases are mesophilic and exhibit high thermostability at 40–60 °C [20,22,24]. These well-characterized chitinases are generally active against colloidal chitin, a type of chitin comprised of amorphous and relatively crystalline parts. In nature, chitin is organized in highly insoluble crystalline arrangements; therefore, chitinase, which can efficiently hydrolyze crystalline chitin, has a better application potential in preparing COSs [1,3,4,5,6]. So far, a few crude enzymes produced by wild strains and some recombinant chitinases have been reported to directly degrade crystalline chitin [14,25,26,27].

The genus *Shewanella* contains typical bacteria belonging to the family Shewanellaceae within the class Gammaproteobacteria. The potential for chitin degradation at the species level in *Shewanella* has been reported; for example, Zou et al. found that *Sh. baltica* 139 and *Sh. putrefaciens* MA h5 could not only secrete two types of exochitinases but also produce endochitinase [28]. Raimundo et al. reported that *Shewanella* spp. from the octocoral *Eunicella labiata* and sponge *Sarcotragus spinosulus* can degrade colloidal chitin in vitro [29]. Nevertheless, few studies have reported the expression and biological functions of chitinase-encoded genes from *Shewanella*. In this study, after a genomic analysis of *Sh*. *khirikhana* JW44 to identify genes related to chitin degradation, the function of *SkChi65* was investigated as a novel chitinase that can facilitate the bioconversion of chitin.

## 2. Materials and Methods

### 2.1. Strain Source, Genome Sequencing, and Functional Annotation

Strain JW44, isolated from the intestines of healthy shrimp *Litopenaeus vannamei* in Zhanjiang, China, grew well at 30 °C when colloidal chitin (2% *w*/*v*) was used as the sole carbon source, supplemented with (NH_4_)_2_SO_4_ (0.1%), KH_2_PO_4_ (0.02%), K_2_HPO_4_ (0.16%), MgSO_4_ (0.02%), NaCl (0.5%), FeSO_4_ (0.001%), CaCl_2_ (0.002%), and agar (1.5%). The colloidal chitin used in this study was prepared from chitin powder (Shanghai Yuanye Biotechnology Co., Ltd., Shanghai, China) according to the method described by Lee et al. [30]. Routine cultivation and preservation, genome sequencing, gene prediction, and functional annotation of JW44 were performed as previously described by Xue et al. [31]. The complete genome sequence of JW44 has been deposited in the NCBI database with accession numbers CP143082 for one chromosome and CP143083 for one plasmid. JW44 was identified as *Sh. khirikhana* based on the analysis of the average nucleotide identity of the genome sequence.

### 2.2. Analysis of Genes Associated with Chitin Degradation

To explore potential genes involved in chitin degradation, the annotated proteome file of JW44 was subjected to analyses in the online STRING website (https://cn.string-db.org/, accessed on 10 December 2023); then, the functional protein association network of predicted genes was constructed based on the output file using Cytoscape 3.9.0 software [32]. The molecular masses of the predicted proteins of the six candidate genes were calculated using the ExPASy Proteomics Server (http://ca.expasy.org/, accessed on 15 December 2023). Functional domain prediction of these genes was performed using SWISS-MODEL (http://www.swissmodel.expasy.org/, accessed on 18 December 2023) and visualized using the IBS online mapping tool (https://ibs.renlab.org/#/server, accessed on 18 December 2023).

### 2.3. Gene Cloning

Of the six candidates, a predicted chitinase gene, *JW44_02465*, renamed *SkChi65*, was selected for cloning, expression, and subsequent analyses. Based on the genome sequence of JW44, the primers *SkChi65*-F (5′-ACAAGGCCATGGCTGATATCATGAAACAACCCCCCTACAGCG-3′) and *SkChi65*-R (5′-GGATCTCAGTGGTGGTGGTGGTGGTGCTCGAGGATGTTGCAGACAAAGTCCCAATC-3′) were designed to amplify *SkChi65* from JW44 genomic DNA as described by Xue et al. [24]. The target gene was cloned into the EcoRV and Xhol sites of the vector pET-32a. Then, the recombinant plasmid was transformed into *Escherichia coli* BL21 (DE3) for the expression of SkChi65.

### 2.4. Bioinformatic Analysis

Based on the gene-cloning results, the amino acid sequence of SkChi65 was deduced using ORF Finder (https://www.ncbi.nlm.nih.gov/orffinder/, accessed on 20 December 2023). The Conserved Domain Database (http://www.ncbi.nlm.nih.gov/Structure/cdd/wrpsb.cgi, accessed on 22 December 2023) was used to predict the typical domains. Secondary structure prediction was performed using SMART (https://smart.embl.de/, accessed on 22 December 2023), multiple sequence alignment was performed using DNAMAN 9.0, and the phylogenetic tree of SkChi65 with the most similar homologs was constructed using MEGA 11.0 by the neighbor-joining method [33]. Analysis and visualization of tertiary structures were performed using Alphafold2 (https://colab.research.google.com/github/sokrypton/ColabFold/blob/main/AlphaFold2.ipynb#scrollTo=kOblAo-xetgx, accessed on 25 March 2024) and PyMOL3.11.

### 2.5. Optimization of Expression

The recombinant *E. coli* BL21 (DE3) was cultured in LB medium at 37 °C for 2–4 h until the OD_600_ was 0.5. The effects of temperature, concentration of isopropyl-β-d-thiogalactopyranoside (IPTG), and induction time on optimal expression of SkChi65 were investigated per the procedures described by Xue et al. [24], with temperatures at 16 °C, 25 °C, and 37 °C, IPTG concentrations at 0, 0.2, 0.4, 0.6, 0.8 and 1.0 mM, and induction times of 0, 2, 4, 6, and 8 h, respectively. After cultivation, the bacterial solution was centrifuged at 8000× *g* for 10 min at 4 °C, the pellet was rinsed with PBS, then mixed with 100 μL PBS and 20 μL protein-loading buffer, and the mixture was checked by SDS-PAGE after being boiled for 10 min.

### 2.6. Analysis of Solubility and Purification

For solubility analysis, after 6 h of incubation of *E. coli* BL21 (DE3) containing the recombinant plasmid under optimal conditions with shaking, a 50 mL sample was centrifuged at 3000× *g* for 10 min. The cell pellet was collected, resuspended, and dispersed using sonification (on for 5 s, off for 8 s, amplitude setting of 30%, and crushing for 30 min). After centrifugation, the supernatant was analyzed directly by SDS-PAGE and used as a crude enzyme extract for purification; the pellet was incubated with 8 M urea overnight and then also subjected to SDS-PAGE after centrifugation [24]. For the purification of SkChi65, the 2 mL resin bed Ni-NTA affinity column was washed with 2 mL of PBS five times. The crude extract of SkChi65 was mixed with the column and incubated on ice for 2 h. The column was washed with 2 mL of imidazole elution at 2, 4, 10, and 20 mM orderly (20 mM sodium phosphate, 300 mM sodium chloride, pH 7.4) to remove miscellaneous proteins. After that, the column was washed with 2 mL of imidazole at 100 mM four times. Then, with 2 mL of imidazole at 250 mM four times, all the eluents were collected as enzyme fractions and verified by SDS-PAGE.

### 2.7. Enzyme Activity Assay

The chitinase activity was determined according to the method described by Imanaka et al. [34] with minor modifications of 3, 5-dinitrosalicylic acid used as a color reagent and absorbance measured at 540 nm. Briefly, the reaction mixture, containing 450 μL of colloidal chitin or other substrates (1%, *w*/*v*) and 50 μL of moderately diluted enzyme solution, was incubated at 37 °C at pH 7.0 for 1 h. The supernatant of the mixture was obtained after centrifugation at 5000× *g* for 5 min at 4 °C; then, 250 μL of 3,5-dinitrosalicylic acid (7.5 mg/mL) was added to the supernatant and heated in boiling water for 10 min. The residual chitin was removed by centrifugation at 12,000× *g* for 5 min. The OD_540_ of the supernatant was measured after cooling. One unit of chitinase activity was defined as the amount of enzyme required to produce 1 μmol GlcNAc per minute under the assay conditions. Assays were carried out in triplicate, and the results are expressed as mean ± standard deviation. The specific activity of SkChi65 is expressed in units per milligram of protein. The protein concentration was determined by the Bradford method using bovine serum albumin as the standard [35].

### 2.8. Biochemical Characterization

The optimal temperature and pH of SkChi65 were determined by measuring its activity, as described in Section 2.7 with modifications of incubation with the substrate at temperatures ranging from 4 °C to 70 °C and at pH ranging from 3.5 to 10.0, respectively; 50 mM of each buffer were used: acetate buffer (pH 3.5–5.5), sodium citrate–phosphate buffer (pH 5.5–7.0), Tris-HCl buffer (7.0–9.0), and glycine buffer (pH 8.0–10.0) [24]. The highest activity of SkChi65 at 40 °C and pH 7.0 was considered 100%. For the thermostability assay, the SkChi65 was pre-incubated without substrates at pH 7.0 for 1 h at temperatures from 4 °C to 70 °C; then, the remaining activity was measured as described in Section 2.7. For pH stability, the SkChi65 was pre-incubated without substrates at 40 °C for 1 h at pH from 3.5 to 10.0, followed by activity measurement. As the control, the activity of SkChi65, with no pre-incubation, was immediately measured and considered 100%. All experiments were repeated at least three times. For the determination of enzyme kinetics, the solution of purified SkChi65 was incubated with colloidal chitin at concentrations of 0.5, 1, 2, 6, 8, 10, 12, 14, 16, and 20 mg/mL in 50 mM Tris-HCl buffer (pH 7.0) at 37 °C for 10 min. The enzyme activity was measured as described above; then, the kinetic parameters of *K_m_*, *V_max_*, *K*_cat_, and *K*_cat_/*K*_m_ were calculated based on the Lineweaver–Burk double reciprocal plotting method.

To investigate substrate specificity, the activities of purified SkChi65 (50 μL, 0.041 mg/mL) towards various substrates, including colloidal chitin, chitin powder, glycol chitosan (60% deacetylation degree [DD]), chitosan (80% DD), carboxylated chitosan (90% DD) (Shanghai Aladdin Biochemical Technology Co., Ltd., Shanghai, China), carboxylmethyl cellulose, and microcrystalline cellulose (450 μL, 10 mg/mL) (Sinopharm Chemical Reagent Co., Ltd., Shanghai, China), were assayed as described in Section 2.7.

### 2.9. Analysis of Hydrolysis Products from Chitooligomers

To reveal the mode of action, time courses of chitooligomer ((GlcNAc)_2–6_) hydrolysis were performed chronologically for 24 h and visualized by silica gel thin-layer chromatography (TLC) according to the method of Imanaka et al. [34] with modifications. The reaction mixture (160 μL) containing chitooligomers (chitobiose–chitohexaose, 1 mg/mL) and 32.8 nM of SkChi65 (0.041 mg/mL) was incubated at 40 °C for 0, 1, 5, 15, 60, 720, and 1440 min at pH 7.0. Subsequently, the reaction products were spotted onto a silica gel plate after being boiled for 15 min; then, the plate was developed for 1 h with *n*-butyl alcohol–acetic acid–water–ammonia (10:5:5:1, *v*/*v*/*v*/*v*). To visualize the final products, the plate was sprayed with a reagent of diphenylamine–aniline–phosphate (0.8 g diphenylamine, 40 mL acetone, 0.8 mL aniline, and 4 mL of 85% phosphoric acid) and heated at 120 °C for 5 min.

## 3. Results

### 3.1. Analysis of Chitin-Utilization Genes in the JW44 Genome

**Figure 1:**
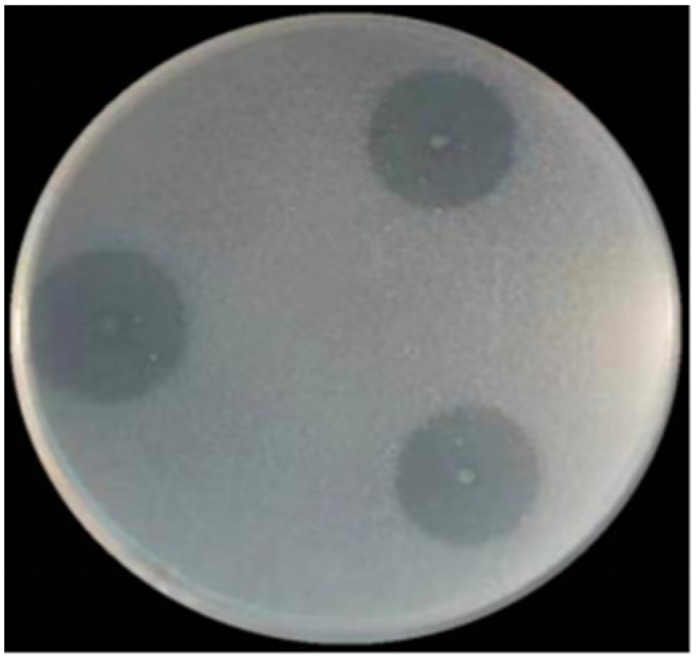
Hydrolysis halo of *Sh. khirikhana* JW44 on agar plate containing 2% colloidal chitin after 5 days of culture.

**Figure 2:**
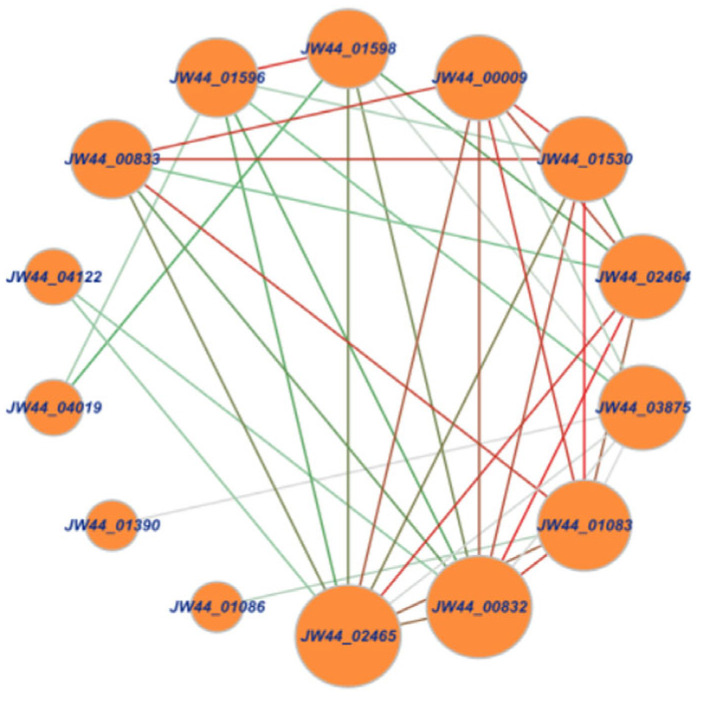
Interaction network of chitin-degradation-related genes of *Sh. khirikhana* JW44. Note: nodes and lines represent genes and interactive relationships between genes, respectively. The size of a node indicates its importance. As for the lines from green to red, the darker the color, the closer the interaction is.

After culturing for 5 days, clear zones on the agar plates were produced by JW44 when colloidal chitin was used as the sole carbon source (Figure 1), indicating that JW44 produced high extracellular chitinolytic activity. Furthermore, based on the genome sequence, 14 genes were annotated as encoding proteins functionally related to chitin utilization, and there were remarkable interactions among these genes (Figure 2). Except for *JW44_04122*, *JW44_04019*, *JW44_01390*, and *JW44_01086*, the other ten genes demonstrated significant roles in the gene interaction network; in particular, *JW44_02465* and *JW44_00832* may be more crucial because of their larger node sizes and darker line colors. Of these, six genes may be directly related to the degradation of long-chain chitin to oligomers, whereas the other four genes are mainly associated with chitin oxidation (*JW44_02464*) or metabolism of the monomer GlcNAc (*JW44_03875*, *JW44_01598*, and *JW44_01596*).

**Figure 3:**
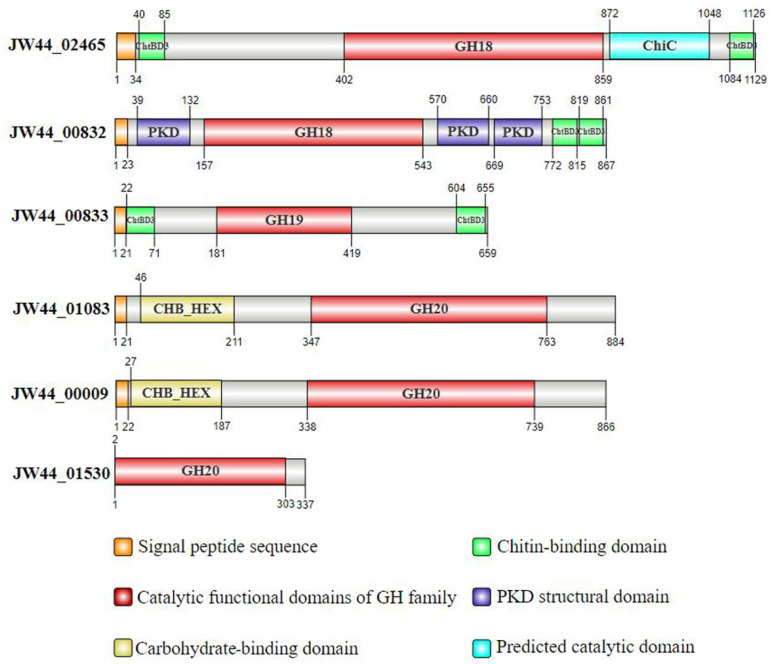
Structure domain predictions of six enzymes encoded by chitin-degradation-related genes derived from *Sh. khirikhana* JW44.

Table 1 lists the genomic and enzymatic information of the six genes mentioned above. These genes mainly belong to GH families 18, 19, and 20; in particular, genes *JW44_02465* and *JW44_00832* differed in size and EC number, although they both were annotated as chitinase A. *JW44_01083* and *JW44_01530* also differed in size, even though they both were assigned as β-*N*-acetylhexosaminidases. Figure 3 further displays the diverse functional domains of the six genes. Notably, they all harbored catalytic domains of GH families; three genes, including two GH18 family genes and one GH19 family gene, possessed two chitin-binding domains (i.e., ChtBD_3_) that may be conducive to the binding of the enzyme to substrate.

### 3.2. Phylogeny of SkChi65 with Other Chitinases

**Figure 4:**
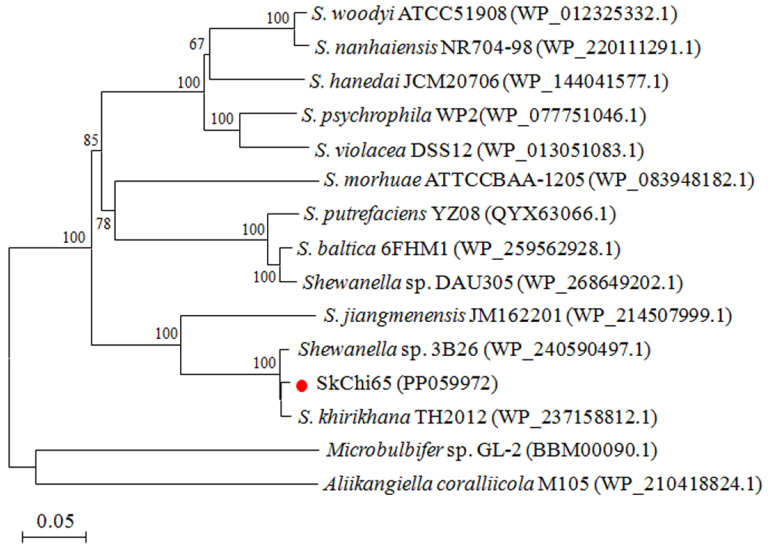
Phylogenetic tree of SkChi65 and other chitinases of the GH18 family from *Shewanella* constructed using the neighbor-joining method. Note: Chitinases from *Microbulbifer* sp. GL-2 and *Aliikangiella coralliicola* M105 were used as an outgroup. Bootstrap analysis of 1000 replicates was conducted, and values above 50% are shown. The SkChi65 in this study is indicated by red dot. The scale bar indicates the amount of genetic change measured as the number of amino acid substitutions per site.

Considering the significant role of *JW44_02465* in the interaction network, this gene, renamed *SkChi65*, was confirmed to have a size of 3390 bp after gene cloning. Protein sequence analysis showed that SkChi65 may be a member of the GH18 family because it harbors the catalytic domains Glyco_18 and ChiC and two chitin-binding domains (ChtBD_3_). Figure 4 shows the phylogenetic tree of SkChi65 with the most similar homologs of chitinases derived from species of the genus *Shewanella*. SkChi65 has the highest amino acid identity (98.58%) with chitinase from *Shewanella* sp. 3B26 and >80% identity with homologs from *Sh. khirikhana* TH2012 and *Sh*. *jiangmenensis* JM162201, and these four chitinases cluster closely into one branch.

**Figure 5:**
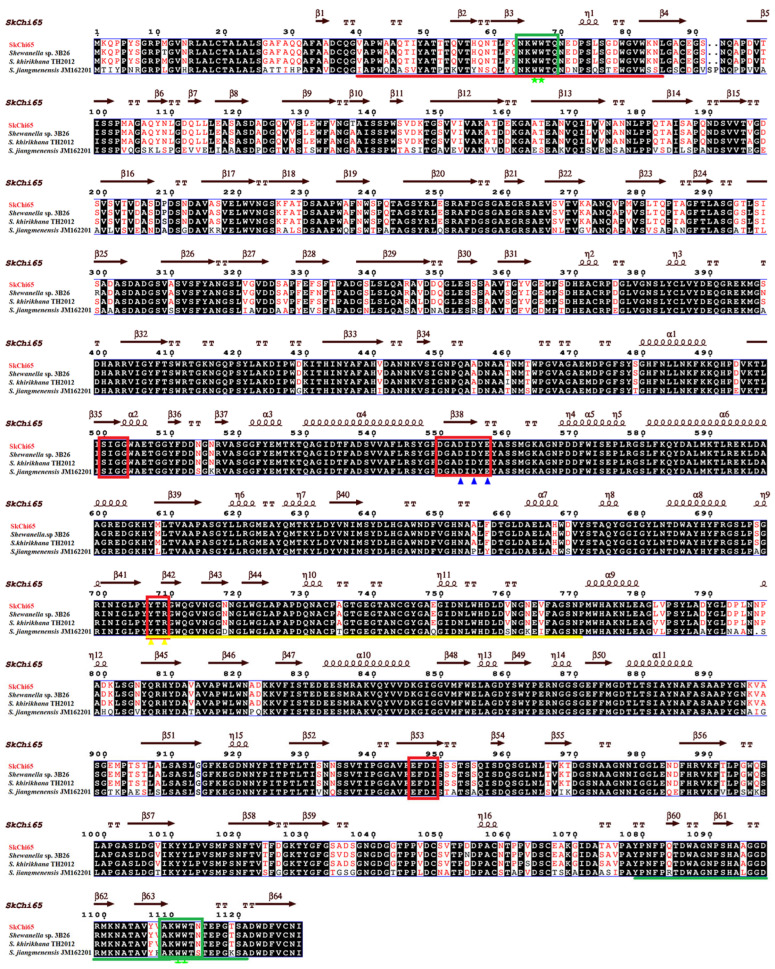
Multiple sequence alignment of SkChi65 from *Sh. khirikhana* JW44 with the most similar chitinases from *Shewanella* sp. 3B26 (WP 240590497), *Sh. khirikhana* TH2012 (WP 237158812), and *Sh. jiangmenensis* JM162201 (WP 214507999). Note: helices are indicated by springs, strands by arrows, and turns by TT letters. Identical residues are shown in white on a black background, and similar residues are shown in red on a white ground. The conserved catalytic motifs are boxed in red with key residues Glu (E) and Asp (D) marked by blue triangles. The typical substrate-binding motifs are boxed in green with two aromatic residues Trp (W) marked by green stars. The chitinase insertion domain (CID) is underlined in yellow with Tyr (Y) and Arg (R) marked by yellow triangles. The two chitin-binding domains (ChtBD_3_) are underlined in red and green, respectively.

**Figure 6:**
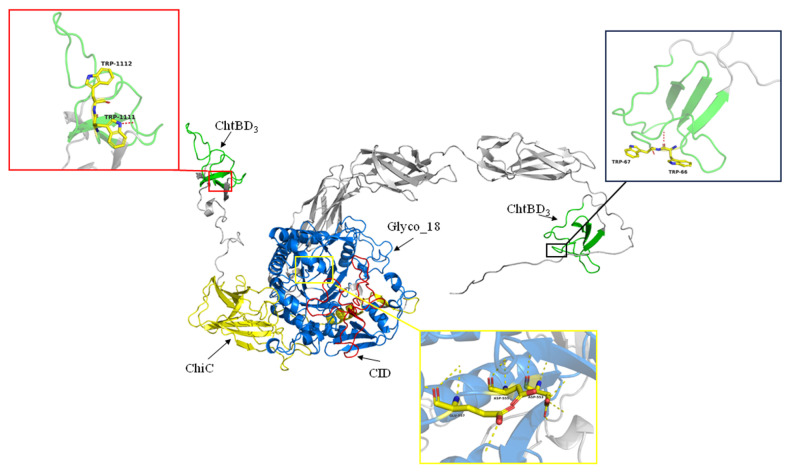
Tertiary structure prediction of protein SKChi65 with the CID (chitinase-insertion domain), ChtBD_3_ (chitin-binding domain), Glyco_18, and ChiC (catalytic domains), active amino acids of TRP in ChtBD_3_ and of ASP in CID indicated.

Structurally, the catalytic domain of SkChi65 possesses four conserved motifs, DxxDxDxE, SxGG, YxR, and [E/D]xx[V/I]. In particular, the YxR motif located in the CID is important for substrate binding and catalysis. Furthermore, the [A/N]KWWT[N/S/Q] motif was found in both ChtBD_3_, and two consecutive aromatic residues were found not only in the N-terminal ChtBD_3_ (Trp^66^ and Trp^67^) but also in the C-terminal ChtBD_3_ (Trp^1111^ and Trp^1112^), which may play crucial roles in chitin binding (Figure 5). A model for the three-dimensional (3D) structure of SkChi65 visually displays these catalytic and chitin-binding domains, especially the (α/β)_8_-TIM barrel, and CID represent key catalytic positions (Figure 6).

### 3.3. Optimization of SkChi65 Expression

**Figure 7:**
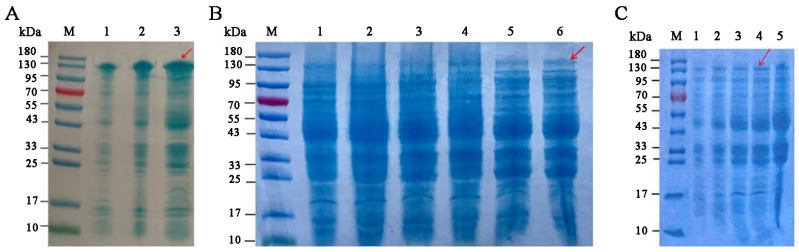
Optimization of expression of recombinant SkChi65. Note: Lane M: protein molecular mass marker; Lanes (**A**) 1–3: induction temperatures of 16, 25, and 37 °C, respectively; Lanes (**B**) 1–6: IPTG induction concentrations of 0, 0.2, 0.4, 0.6, 0.8, and 1.0 mM, respectively; Lanes (**C**) 1–5: induction times of 0, 2, 4, 6, and 8 h, respectively. The target enzyme bands are indicated by red arrows.

Figure 7 shows the expression levels of recombinant SkChi65 at different temperatures, IPTG induction concentrations, and induction times. First, Figure 7A,B demonstrates that the expression of SkChi65 was highest at 37 °C and 1.0 mM IPTG, respectively. Under these conditions, a relatively higher expression was observed at 6 h. Thus, a 6 h induction with 1.0 mM IPTG at a growth temperature of 37 °C was appropriate for recombinant SkChi65 to be optimally expressed in *E. coli* BL21 (DE3) (Figure 7C).

### 3.4. Solubility and Purification of SkChi65

**Figure 8:**
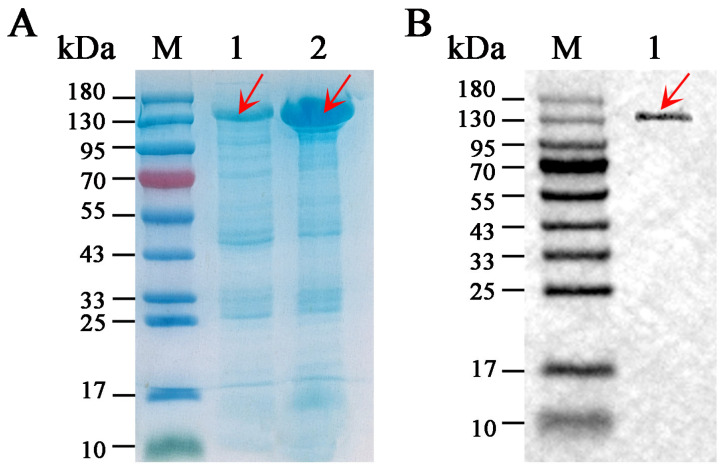
SDS-PAGE analysis of recombinant SkChi65 from *E. coli* BL21 (DE3) cells. Note: Lane M, protein molecular mass marker; Lanes (**A**) 1–2: crude extract from lysate precipitate and lysate supernatant of *E. coli* containing recombinant SkChi65; Lane (**B**) 1: purified SkChi65. The target enzyme bands are indicated by red arrows.

As shown in Figure 8A, the content of SkChi65 expressed in the supernatant of *E. coli* lysate was much higher than that in the pellets, indicating that *SkChi65* was successfully expressed in *E. coli* and that SkChi65 was mainly soluble and relatively less found in inclusion bodies. The soluble protein was purified via His-tag affinity chromatography, and an apparently homogeneous band of approximately 140 kDa was observed on SDS-PAGE gel, which is in accordance with the molecular mass of 139.95 kDa predicted from its sequence (Figure 8B).

### 3.5. Enzymatic Properties of SkChi65

**Figure 9:**
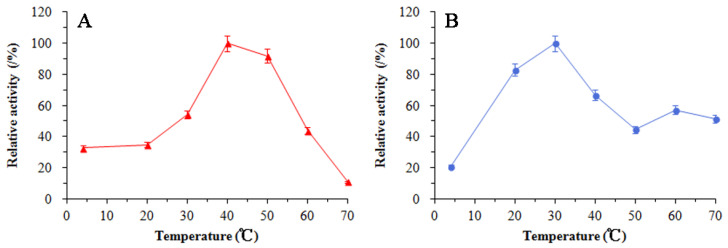
Effect of temperature on the activity (**A**) and the stability (**B**) of SkChi65.

**Figure 10:**
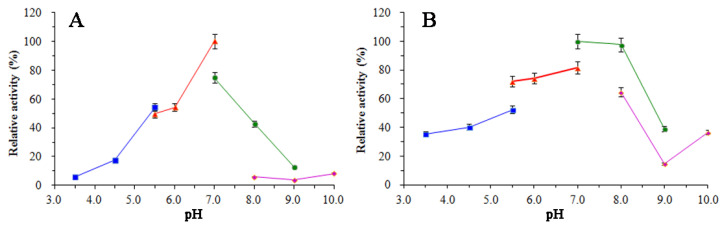
Effect of pH on the activity (**A**) and the stability (**B**) of SkChi65. Bule line represents acetate buffer (pH 3.5–5.5); red line represents sodium citrate-phosphate buffer (pH 5.5–7.0); green line represents Tris-HCl buffer (7.0–9.0); purple line represents glycine buffer (pH 8.0–10.0).

As shown in Figure 9A, SkChi65 exhibited the highest activity towards colloidal chitin at 40 °C and relatively high activity at 50 °C. Regarding thermal stability (Figure 9B), SkChi65 retained ~100% activity at 30 °C after 1 h incubation, which was greater than the 80%, 60%, and 40% activities at 20, 40, and 50–70 °C, respectively. Collectively, these data suggest that SkChi65 is fairly thermostable with a temperature optimum of 40 °C. Regarding pH dependency, Figure 10A shows that SkChi65 exhibited the highest activity at pH 7.0. Activity dropped steeply below pH 5.5 or above pH 8.0. The highest pH stability of SkChi65 was achieved at pH 7.0 in Tris-HCl buffer (Figure 10B). SkChi65 also maintained higher than 90% activity at pH 8.0 and even maintained more than 70% activity at pH 5.5–7.0 after incubation for 1 h. Collectively, these data indicate that SkChi65 has a neutral pH optimum and is highly stable from pH 5.5 to 8.0.

**Figure 11:**
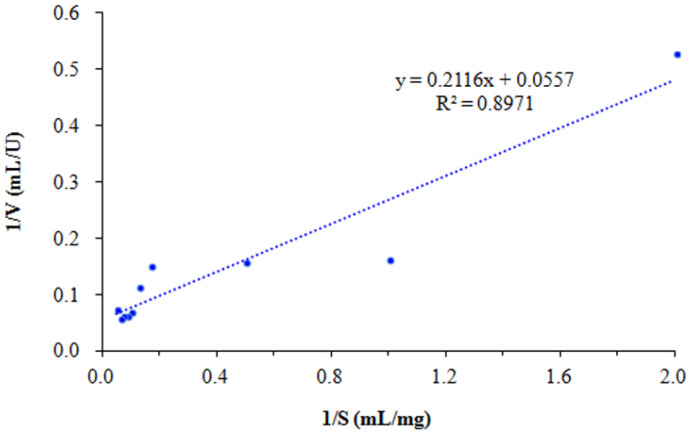
The Lineweaver–Burk double inverse curve of SkChi65 on colloidal chitin. Note: V: the rate of reaction, S: the concentration of colloidal chitin.

Based on the results of SkChi65 activity measured at different colloidal chitin concentrations, the kinetic parameters *K_m_*, *V_max_*, and *kcat* were calculated to be 27.2 μM, 299.2 μMs^−1^, and 10,203 s^−1^, respectively, according to the Lineweaver–Burk double inverse curve (Figure 11), and the value of k*cat*/*K_m_* was 375.1 μM^−1^s^−1^, indicating a high affinity and catalytic efficiency of SkChi65 for colloidal chitin.

### 3.6. Analysis of Substrate Specificity and Hydrolysis Product

Table 2 shows that SkChi65 had the highest chitinase activity towards colloidal chitin, followed by glycol chitosan (60% DD) and chitin powder. Relatively weak activities were observed when chitosan (80% DD) and carboxylated chitosan (90% DD) were used as substrates, and no chitinase activity was detected in the presence of carboxymethyl cellulose and microcrystalline cellulose. Thus, SkChi65 was active on crystalline chitin and could cleave the β-1,4-glycoside bond between monomers of GlcNAc or GlcN, but not glucose.

**Figure 12:**
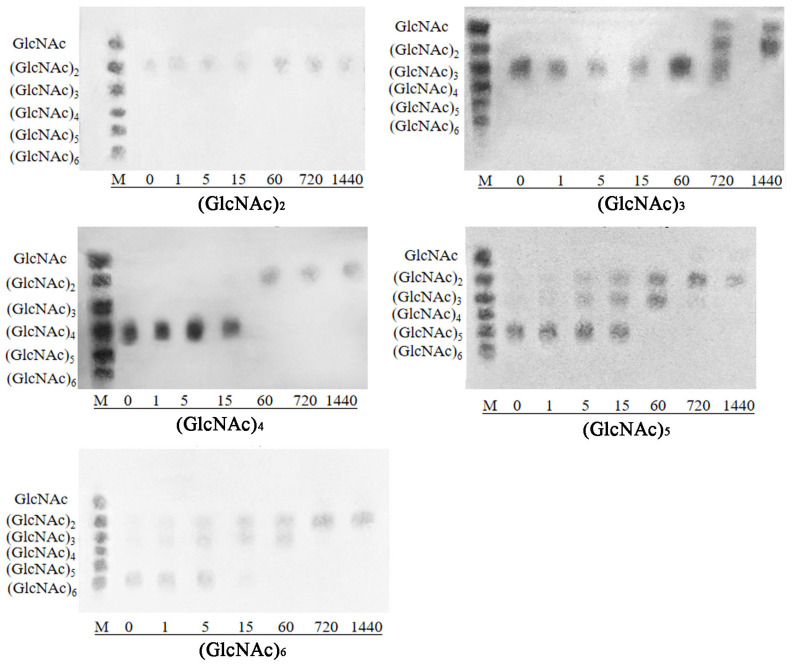
Thin layer chromatography analysis of hydrolytic products of chitooligomers catalyzed by purified SkChi65. Note: Lane M: standard *N*-acetyl chitiooligosaccharides ranging from (GlcNAc)_2_ to (GlcNAc)_6_. The time of incubation (in min) and the tested substrate are given below each panel.

As shown in Figure 12, TLC analysis revealed that chitotriose (GlcNAc)_3_ was hydrolyzed by SkChi65 to chitobiose (GlcNAc)_2_ and GlcNAc within 24 h, whereas (GlcNAc)_2_ was not degraded. Chitotetraose (GlcNAc)_4_ was hydrolyzed to (GlcNAc)_2_ in 1 h; (GlcNAc)_5_ was hydrolyzed to (GlcNAc)_2_ and GlcNAc, with (GlcNAc)_3_ observed as an intermediate product. Therefore, SkChi65 tended to act as an exo-type chitinase with (GlcNAc)_2_ as the main hydrolysis product. Meanwhile, (GlcNAc)_6_ was first hydrolyzed into (GlcNAc)_2_ and (GlcNAc)_3_. This indicated that the third glycosidic bond of (GlcNAc)_6_ might be cut by SkChi65, so it may be also an endochitinase.

## 4. Discussion

Although chitin can be utilized by numerous bacteria, genome-wide investigations have been conducted mainly in vibrios [2,36,37,38]. In this study, the genome of *Sh. khirikhana* JW44 was predicted to possess a core set of genes responsible for chitin utilization. The potential functions of these annotated genes were similar to those proposed in the family Vibrionaceae by Meibom et al. [2] and Hunt et al. [36]. Except for genes related to deacetylation and oxidation of chitin, JW44 possessed six genes involved in chitin degradation. Presumably, chitinase A may begin to degrade chitin polymers into oligomers outside of the bacteria, which can be transported into the periplasmic space to be degraded into (GlcNAc)_1–2_ by chitodextrinase and β-*N*-acetylhexosaminidase. Then, (GlcNAc)_1–2_ can be transported into the cytosol and metabolized by chitobiase along with other enzymes. Therefore, JW44 harbors the complete equipment needed for the degradation of chitin and its derivatives. However, some enzymes, including chitinase A and β-*N*-acetylhexosaminidase, are encoded by more than one gene, thus suggesting gene versatility in the chitin degradation pathway, although the chitinolytic ability is considered to be highly conserved across diverse bacterial genera [29,37,38].

Next, the chitinase gene *SkChi65* was successfully cloned and heterologously expressed; its predicted amino acid sequence was most similar to that of the glycoside hydrolase from *Shewanella* sp. 3B26 (98.58%, WP 240590497). SkChi65 was assigned as one member of the GH18 family with modular structures, including a Glyco_18 catalytic domain (an (α/β)_8_-TIM barrel), a ChiC core domain, and two chitin-binding domains (ChtBD_3_) [17,39]. In the catalytic domain of SkChi65, the typical motifs DxxDxDxE and SxGG were found, which are perfectly conserved across bacteria, fungi, archaea, and invertebrates. The Glu and Asp residues contained in these motifs may be involved in proton donation and glycosidic bond breakage [19,39,40,41,42,43,44,45].

Similar to that reported by Li and Greene [12], SkChi65 has a CID (α+β insertion domain) between amino acids Y^707^ and R^771^, which classifies SkChi65 into subfamily A of the GH18 family of processive exochitinases. The CID is embedded in the catalytic domain between the seventh β-strand and the α-helix of the (α/β)_8_-TIM barrel. The conserved motif YxR, along with motif [E/D]xx[V/I] in the catalytic domain may play key roles in the interaction with polysaccharides. Lee et al. also suggested that domains of the (α/β)_8_-TIM barrel and CID could facilitate chitinase-substrate binding via the formation of a deep substrate-binding cleft [39]. Notably, Zees et al. reported that deletion of the CID of ChiA from *Serratia marcescens* led to a shallower tunnel in the catalytic domain of the mutant than that of the intact enzyme, which was further accompanied by substantially reduced thermal stability, specific activity, and altered substrate specificity [18]. Therefore, these conserved motifs and domains play crucial roles in chitinase catalytic activity.

Generally, chitinases have only one binding domain that exists either at the C-terminus or N-terminus [14,17,43]. However, two ChtBD_3_ were identified in SkChi65, with one at the C-terminus and the other at the N-terminus. The Pfam database categorizes ChtBD_3_ as either CBM5 or CBM12 (carbohydrate-binding module). The CBM5 of SkChi65 showed high identity with different sequences from strains of the genus *Shewanella* deposited in the NCBI database. The consensus sequence AKWWTK, which is well conserved in the CBM5 modules in bacteria and archaea, also appeared to be moderately conserved in *Sh. khirikhana* JW44, and three aromatic amino acids (Y^49^W^66^W^67^ in the N-terminus and Y^1079^W^1111^W^1112^ in the C-terminus) are conducive to the binding of enzyme and substrate [39,43,46]. Therefore, these ChtBD_3_ in chitinases may greatly facilitate the interaction between enzyme and substrate via aromatic residues binding to the sugar molecules.

Among the substrates of chitin and its derivatives, purified SkChi65 exhibited the highest specific activity towards colloidal chitin, which was also higher than that of some reported chitinases [14,41]. Interestingly, SkChi65 exhibited excellent activity towards glycol chitosan (DD 60%) and crystalline chitin and even showed relatively good activity towards chitosan (DD 80%) and carboxylated chitosan (DD 90%). These activities are distinct from those of many chitinases, such as Chi23 of *Pseudoalteromonas aurantia* DSM6057, which exhibits no activity towards glycol chitosan and chitosan [20], and *Vh*ChiA from *Vibrio harveyi*, which cannot bind to chitosan [17]. Cloning and structural analysis of the gene fragment encoding the chitin-binding domain of *Vh*ChiA by Suginta et al. indicated that the tandem *N*-acetamido of the chitin chain functions as the specific site of enzyme–substrate interaction [17], which contributes to the affinity for chitin but not chitosan. This result was confirmed by the present study. The higher the degree of deacetylation, the lower the activity that was observed for the three kinds of chitosan, with degrees of deacetylation ranging from 60% to 90%.

In addition to the catalytic domain, these results may be attributed to the two ChtBD_3_ of SkChi65 with conserved surface-exposed aromatic residues, which are essential to the interaction of chitinase with insoluble chitin [19,46,47]. Zhao et al. reported that the high chitin-binding capacity of chitinase ChiC8-1 from *Chitinilyticum* sp. C8 may be related to its two chitin-binding domains, both at the N-terminus [48]. Uni et al. reported that the chitin binding by Trp^541^ and Trp^542^ in an alkaline chitinase from *Bacillus* sp. J813 improves the hydrolysis of insoluble chitin but not of soluble chitin [49]. When crystalline chitin was used as a substrate, Juárez-Hernández et al. reported that ChiA74 of *Bacillus thuringiensis* exhibited a significantly lower activity after the deletion of the CBD/FnIII domains [19]. Compared to chitin powder, glycol chitosan, with certain degrees of deacetylation, is more water soluble, which may increase the local concentration of the enzyme around the substrate and induce efficient hydrolysis; however, the exact mechanism by which SkChi65 binds to glycol chitosan remains to be elucidated.

Various physicochemical factors affect the industrial applications of chitinases, among which temperature and pH are particularly important for enzyme activity and stability. In this study, chitinase SkChi65 exhibited the highest activity at 40 °C and at pH 7.0, retaining approximately 50% activity after incubation for 1 h at 20–70 °C or pH 5.5–8.0. This indicates that this enzyme is pH neutral and mesophilic, which is similar to that of many bacterial chitinases with optimal temperatures at 40–60 °C and pH optima of 6.5–7.5 [23,50].

Parameter *K_m_* reflects the affinity of the enzyme for different substrates, and the smaller the *K_m_* is, the higher the affinity of the enzyme for the substrate. The *K_m_* of SkChi65 for colloidal chitin was 27.2 μM, i.e., 3.80 mg/mL, which was significantly lower than that of most bacterial chitinases, such as 9.28 mg/mL for MtCh509 from *Microbulbifer thermotolerans* DAU221 [39] and 47.92 mg/mL for SmChiD from *Se*. *marcescens* GPS5 [51], suggesting a higher binding affinity of SkChi65 towards colloidal chitin and providing good potential for functional applications. The *k_cat_* indicates the catalytic efficiency of the enzyme, although the *k_cat_* (10,203 S^−1^) of SKChi65 was remarkably lower than the 27,334 S^−1^ of ChiA-Si40 from *Sh. inventionis* HE3 [52]. However, it was obviously higher than the 5000 S^−1^ of ChiA-65 from *Bacillus licheniformis* LHH100 [53] and 19.1 S^−1^ for chitinase B from *Serratia marcescens* [54]. For the latter, Vaaje-Kolstad et al. [55] reported that the *k_cat_* of ChiB from *Se*. *marcescens* greatly dropped to 0.26–0.34 s^−1^ when the mutation of Asp^142^ to Asn occurred in the conserved sequence motif of D^140^xD^142^xE^144^, indicating the importance of electrostatic interaction with Asp^142^ for the enzymatic affinity. Further, the level of *k_cat_*/*K_m_* of SkChi65 was up to 375.1 μM^−1^s^−1^, which was significantly higher than 0.56 of ChiB from *Se. marcescens* towards oligomers [55]. These results indicated that SkChi65 harbors a high catalytic capacity to colloidal chitin.

When chitooligomers (GlcNAc)_2–6_ were used as substrates, the major hydrolysis products of SkChi65 were (GlcNAc)_2_ and GlcNAc, which were identical to those of some reported chitinases, such as MtCh509 from *M. thermotolerans* DAU221 [39] and PxChi52 from *Paenibacillus xylanexedens* Z2-4 [56]. However, SkChi65 could not utilize (GlcNAc)_2_ as a substrate. Similarly, ChiC8-1 isolated from *Chitinilyticum* sp. C8, FbalChil8A from *Ferrimonas balearica,* and MvarChi18A from *Microbulbifer variabilis*; all could not use (GlcNAc)_2_ [48,57]. Hence, considering the principal product of (GlcNAc)_2_, SkChi65 may be an exochitinase. In addition, the identification of (GlcNAc)_3_ as an intermediate product and GlcNAc as a final product suggests that SkChi65 may also exhibit endochitinase activity. Thus, SkChi65 from *Sh. khirikhana* JW44 could have both exo- and endochitinase activities, which was similar to reports from the other two *Shewanella* species [28].

## 5. Conclusions

In this study, the great chitin-degrading potential of *Sh. khirikhana* JW44 was confirmed by phenotypic and genomic characterizations. A key chitinase-encoding gene, *SkChi65*, was successfully cloned and expressed. The recombinant SkChi65 had a molecular mass of approximately 139.95 kDa, exhibited a wide range of pH dependency (pH 5.5–8.0), thermostability (30–50 °C), and optimal activity at 40 °C and pH 7.0. In addition to colloidal chitin, this enzyme showed activity towards glycol chitosan and chitin powder. Hydrolysis of the chitooligomers (GlcNAc)_2–6_ by SkChi65 produced GlcNAc and (GlcNAc)_2_, as confirmed by TLC. According to the analyses of protein sequence and substrate hydrolysis, SkChi65 is excellent with both exo- and endochitinase activities towards diverse substrates, which may facilitate the bioconversion of chitin waste for industrial applications. Future research aimed at improving and optimizing the production of chitooligosaccharides with this enzyme and/or bacterial strain warrants further investigation.

## Figures and Tables

**Table 1:** Genomic and enzymatic properties of six genes related to chitin degradation of *Sh. khirikhana* JW44.

Locus_Tag	Start	End	Size/aa	Mass/kDa	GH Family	EC Number	Product
JW44_02465	* c2793077	c2796466	1129	139.95	GH18	3.2.1.202	chitinase A
JW44_00832	950609	953212	867	91.32	GH18	3.2.1.14	chitinase A
JW44_00833	953285	955264	659	69.49	GH19	-	chitodextrinase
JW44_01083	1228958	1231612	884	97.20	GH20	3.2.1.52	β-*N*-acetylhexosaminidase
JW44_00009	13742	16342	866	96.14	GH20	3.2.1.52	chitobiase
JW44_01530	1751247	1752260	337	36.36	GH20	3.2.1.52	β-*N*-acetylhexosaminidase

Note: *: “c” denoted the position of the gene on the complement strand of JW44 genome relative to other genes.

**Table 2:** Substrate specificity of SkChi65 from JW44.

Substrates (1%, *w*/*v*)	Specific Activity (U/mg) **	Relative Activity (%)
Colloidal chitin	3.89 ± 2.56	100.0
Glycol chitosan (60% DD *)	2.36 ± 0.57	60.7
Chitin powder	1.24 ± 0.47	31.9
Chitosan (80% DD)	0.63 ± 0.20	16.2
Carboxylated chitosan (90% DD)	0.30 ± 0.20	7.7
Carboxymethyl cellulose	0.00	0.0
Microcrystalline cellulose	0.00	0.0

Note: *: DD (degree of deacetylation); **: data are means ± SD of three replicates.

## Data Availability

The complete genome sequence of *Shewanella khirikhana* JW44 can be found in the NCBI database with accession numbers CP143082 for one chromosome and CP143083 for one plasmid.
